# Dapagliflozin Attenuates Renal Tubulointerstitial Fibrosis Associated With Type 1 Diabetes by Regulating STAT1/TGFβ1 Signaling

**DOI:** 10.3389/fendo.2019.00441

**Published:** 2019-07-03

**Authors:** Fengjuan Huang, Yanyan Zhao, Qingzhu Wang, Jan-Luuk Hillebrands, Jacob van den Born, Linlin Ji, Tingting An, Guijun Qin

**Affiliations:** ^1^Division of Endocrinology, Department of Internal Medicine, The First Affiliated Hospital of Zhengzhou University, Zhengzhou, China; ^2^Division of Pathology, Department of Pathology and Medical Biology, University Medical Center Groningen, University of Groningen, Groningen, Netherlands; ^3^Department of Nephrology, University Medical Center Groningen, University of Groningen, Groningen, Netherlands

**Keywords:** dapagliflozin, tubulointerstitial fibrosis, epithelial mesenchymal transition, STAT1, TGF-β1

## Abstract

Tubulointerstitial fibrosis (TIF) plays an important role in the progression of renal fibrosis in diabetic nephropathy (DN). Accumulating evidence supports a crucial inhibitory effect of dapagliflozin, a SGLT2 inhibitor, on TIF, but the underlying mechanisms remain largely unknown. This study aimed to shed light on the efficacy of dapagliflozin in reducing TIF as well as its possible impact on renal function. TIF in human kidney biopsies obtained from patients with DN was quantified by histopathological staining. *In vitro*, HK-2 cells were incubated in high glucose with dapagliflozin or fludarabine, and epithelial-mesenchymal transition (EMT) was determined. *In vivo* experiments were performed in streptozotocin (STZ)-induced type 1 diabetic mice treated with dapagliflozin by gavage for 16 weeks, after which specific functional characteristics and TIF were analyzed. In both DN patients and diabetic mice, fibronectin and Col IV, as well as STAT1 protein in the kidneys were increased as compared with controls. Dapagliflozin significantly decreased blood glucose, and renal STAT1 and TGF-β1 expression in mice. Furthermore, dapagliflozin improved renal function, and attenuated diabetes-induced TIF. In HK-2 cells, dapagliflozin, and fludarabine directly decreased aberrant STAT1 expression and reversed high glucose-induced downregulation of E-cadherin and α-SMA induction. Thus, the results demonstrate that dapagliflozin not only improves hyperglycemia but also slows down the progression of diabetes-associated renal TIF by improving hyperglycemia-induced activation of the STAT1/TGF-β1 pathway.

## Introduction

Diabetic nephropathy (DN) is one of the most devastating complications of diabetes, and is associated with significant morbidity and mortality in patients with diabetes. According to the International Diabetes Federation, the number of diabetes patients worldwide will increase from 382 million in 2013 to 592 million by 2035 (1). DN is characterized by glomerulosclerosis, overproduction of extracellular matrix (ECM) proteins, tubular atrophy, and tubulointerstitial fibrosis (TIF) (2). While substantial research has focused on glomerular changes in DN, there is a growing body of evidence that shows that tubular lesions and TIF are also important hallmarks of the pathogenesis of DN. TIF represents a common final pathway that leads to progressive renal injury and a better predictor of renal disease progression than glomerular injury (3–5).

Recent studies indicate that renal tubular epithelial-mesenchymal transition (EMT) and ECM accumulation in the renal interstitium play key factors in tubular lesions and TIF in the pathogenesis of DN (6, 7). EMT has been implicated in the accelerated fibrogenesis that is seen in DN (8). In this process, renal tubular cells, in response to injury or local activation, lose their epithelial phenotype and acquire profibrotic features that are characteristic of mesenchymal cells (9). Furthermore, excessive deposition, and dysregulated remodeling of ECM components, such as collagen type IV (Col IV) and fibronectin (FN), are closely related with the pathogenesis and progression of renal fibrosis. Current therapies to prevent DN and slow down its progression is based on glycaemic control, blood pressure control, and RAAS blockade. These therapies are only partially effective, and a substantial residual risk to develop end-stage renal disease still remains (10). Therefore, there is a great demand for new therapies and strategies targeting tubules and the interstitium to retard DN progression.

Sodium glucose co-transporter 2 (SGLT2) is a member of the Slc5 family, and is expressed in the S1 segment of the proximal tubule. Function of this transporter accounts for ~90% of glucose reabsorption in the kidney (11). SGLT2 inhibitors represent a new drug category (12, 13), which reduces renal glucose reabsorption leading to urinary glucose excretion and is thus associated with improvement of glycemic control (12, 14). Among these drugs, orally administered dapagliflozin (DAPA) is a highly selective and potent oral inhibitor of SGLT2, which has been used in patients with type 2 diabetes, improving glycemic control with minimal hypoglycemia, and is also associated with weight loss and systolic blood pressure (SBP) reduction. However, the effects of the SGLT2 inhibitor DAPA on the progression of TIF have not been reported.

We propose that one of the potential renoprotective mechanisms of DAPA might be mediated via the Signal transducer and activator of transcription-1 (STAT1)/ Transforming growth factor-beta 1 (TGFβ1) pathway. The JAK/STAT1 pathway is an essential intracellular mechanism of cytokine actions and constitutes a link between activation of cell-surface receptors and nuclear transcriptional events (15). STAT1, a member of the STAT family of transcription factors, is mainly related to high glucose-induced oxdiative stress and expression of TGF-β1, as well as the production of the ECM proteins collagen IV and FN (16). Meanwhile, high glucose activates STAT1 and TGF β in diabetic nephropathy, and involved in the process of renal tubulointerstitial fibrosis (17). It has been reported that TGF-β isoforms and their receptors are up-regulated in both experimental and human DN (18). TGF-β receptors are serine threonine kinases and they transduce various intracellular signaling when bound by TGF-β. TGF-β1 is one of the most potent growth factor contributing to ECM accumulation, which it does by stimulating ECM production and suppressing its degradation (19). It could be a promising option for preventing loss of renal function and fibrosis in diabetes through Smads dependent or independent pathway (20). Smad proteins are transcription factors that mediate TGF-β/activin signaling by forming complexes with each other (18). Meanwhile, previous studies have demonstrated that the inhibitory effect of suppressor of cytokine signaling-1 (SOCS-1) on high glucose-induced production of TGF-β1 may be via inhibiting the activation of the JAK/STAT signaling pathway (21).

The aim of this study is to demonstrate the renoprotective effects of DAPA on diabetes-induced TIF and study the underlying mechanisms involved. We demonstrate a novel inhibitory role of DAPA on the development of renal TIF in a model of type 1 diabetes in mice *in vivo* and HK-2 tubular epithelial cells *in vitro*. The protective effect is mediated through the STAT1/TGF-β1 signaling pathway. These data suggest that DAPA may be a promising therapeutic agent for the treatment of DN by inhibiting the development of TIF.

## Materials and Methods

### Materials

Rabbit polyclonal antibody for Collagen IV (Cat.No. ab6586) was purchased from Abcam (Cambridge, MA, USA). Anti-FN (Cat.No. 15613-1-AP) was purchased from Proteintech Group (Wuhan, China), a monoclonal antibody for α-SMA was obtained from Novus Biological (Littleton, CO, USA), and a rabbit polyclonal anti-ACTB antibody (Cat.No. D110001) was obtained from Sangon Biotech (BBI, Shanghai, China). Antibodies to STAT1, TGF-β1, and E-cadherin were obtained from Cell Signaling Technology (Danvers, MA, USA). Cy3- and FITC- conjugated goat anti-rabbit secondary antibodies were obtained from BBI Life Science (Shanghai, China). Streptozotocin was purchased from Sigma (St. Louis, MO, USA). Dapagliflozin (DAPA) and STAT1 inhibitor fludarabine (Flu) were procured by MedchemExpress (Shanghai, China).

### Human Kidney Biopsy Studies

Tissue sections from kidney biopsies from patients diagnosed with class III or IV DN (22) and non-diabetic adjacent normal tissues from patients with renal cell carcinoma (NC) were obtained from the first affiliated hospital of Zhengzhou University following the Institutional Review Board of Zhengzhou University. The age and gender were matched between the DN and NC group. The clinical baseline characteristics of subjects included in this study was shown in Table 1. Gene expression was analyzed by quantitative real-time reversed transcriptase polymerase chain reaction (qRT-PCR). Protein expression was analyzed by western blotting and immunohistochemistry. Histochemical stainings include Picrosirius red, Masson's trichrome, hematoxylin and eosin (H&E), and periodic acid-Schiff (PAS) staining. The area of fibrosis in the kidney was determined using Image J software.

**Table 1:** Baseline characteristics of patients with DN and normal controls.

**Characteristic**	**NC (*N* = 10)**	**DN (*N* = 10)**
Sex (M/F)	6/4	5/5
Age (years)	65.3 ± 9.8	67.4 ± 8.6
Scr(umol/L)	72.7 ± 15.4	152.7 ± 35.4^*^
BUN (mmol/L)	4.2 ± 2.6	15.5 ± 2.4^*^
eGFR (ml/min/1.73 m^2^)	102.5 ± 43.5	36.4 ± 20.1^*^
SBP (mm Hg)	121.3 ± 13.5	149.3 ± 18.4^*^
DBP (mm Hg)	72.4 ± 10.3	107.7 ± 14.6^*^

Scr, serum creatinine; BUN, blood urea nitrogen; eGFR, estimated glomerular filtration rate (calculated using the Chronic Kidney Disease Epidemiology Collaboration equation); SBP, systolic blood pressure; DBP, diastolic blood pressure.

* *p < 0.05 as compared to normal controls*.

### Culture and Treatment of Renal Tubular Epithelial Cells

The human renal proximal tubular epithelial HK-2 cell line was obtained from the Cell Bank of Type Culture Collection (Chinese Academy of Sciences, Shanghai, China). HK-2 cells were cultured in Dulbecco's modified Eagle's medium (DMEM) supplemented with 10% fetal bovine serum (Gibco, USA), 1% penicillin (100 U/ml)–streptomycin (100 μg/ml). Cultured cells were incubated in a humidified atmosphere of 5% CO2 and 95% air at 37°C. HK-2 cells at passage 6–10 were used for experiments, and medium was changed every 48 h. The cells were grown to 60–70% confluence and serum-deprived for 24 h before use in experiments. HK-2 cells were treated with normal glucose (NG, 5.6 mM), high glucose (HG, 25 mM), high glucose with either DAPA (5 μmol/L, HG+DAPA-1) or DAPA (10 μmol/L, HG+DAPA-2) or Flu (10 μmol/L, HG+Flu). Cells were collected at 72 h post treatment. Experiments were replicated three times.

### Experimental Animal Model and Treatment

Six-week-old male C57/BL6 mice were purchased from Beijing Vital Rival Laboratory Animal Technology (Beijing, China). The mice were kept on a 12-h light/12-h dark cycle at 23 ± 1°C with 50 ± 10% relative humidity under specific pathogen-free conditions. All animals had free access to drinking water and no adverse effects were observed during the entire duration of the experiment. All experiments were performed according to the Principles of Laboratory Animal Care. Animal experiments were approved by the institutional committees of the Animal Research Committee and Animal Ethics Committee of Zhengzhou University. Diabetes was induced as described previously (23). Briefly, after 12 h of fasting, 6-week-old male C57/BL6 mice received one intraperitoneal injection of 130 mg/kg streptozotocin (STZ) solution in 0.05 M citrate buffer (pH 4.5), a selective β cell genotoxicant, to induce diabetes (24). Blood glucose levels were measured 72 h after STZ injection, which was defined as a fasting blood glucose level higher than 16.7 mmol/L. DAPA (10 mg/kg/day), dissolved and diluted with saline, was administered orally by gavage every day to mice with diabetes for 16 weeks. The drug solution was freshly prepared and changed at least 3 times per week. Diabetic mice (DM) were given saline as vehicle controls. The mice were divided into 5 groups (*n* = 5 per group), and the SGLT2 inhibitor dapagliflozin and saline were administered accordingly: (1) Normal control (NC); (2) DM-8W (Diabetic mice were administered with saline for 8 weeks); (3) DM-12W (Diabetic mice were administered with saline for 12 weeks); (4) DM-16W (Diabetic mice were administered with saline for 16 weeks); (5) DAPA-16W (Diabetic mice were administered with dapagliflozin for 16 weeks).

### Metabolic Analyses

Fasting glucose from mouse tail-vein blood was measured monthly by using a blood glucose meter (ACCU-CHEK Performa; Roche Diagnostics, Mannheim, Germany). Mice were placed in metabolic cages for 24 h urine collection every month. The urine albumin level was measured using an albumin ELISA kit (Bethyl Laboratories, Texas, USA). Body weight was measured monthly. Mice were sacrificed after DAPA treatment for 16W. Blood was collected from the hearts of anesthetized mice into vacuum pro-coagulation tubes (SanLi, China) and was centrifuged at 13,000 r/min for 10 min (Beckman Instruments, Galway, Ireland). Blood urea nitrogen (BUN) and creatinine were measured using ELISA kits (Yaji Biotechnology, Shanghai, China).

### Histopathological Examination

At sacrifice, kidney tissue samples were collected and weighed. Left kidney cortex was fixed in 10% neutral buffered formalin for 24 h, embedded in paraffin, and cut into 3-μm sections. Right kidney cortex was flash-frozen in liquid nitrogen for RNA and protein analyses. To evaluate the effect of DAPA on renal fibrosis, paraffin-embedded tissue sections were deparaffinized and stained with PAS (Jiancheng Bioengineering Institute, Nanjing, China), Masson's trichrome reagent (Solarbio Life Sciences, Beijing, China), Picrosirius red, and H&E (Labio Experimental Audio Supplies, Zhengzhou, China) as previously described (20). Masson's trichrome- and PAS-stained sections were imaged using an optical microscope. A semi-quantitative score for the staining intensity was assessed by examining at least 5 fields in each section using Image J software.

### Immunofluorescence Staining

Immunofluorescence staining of kidney sections was performed as described (25). Initially, kidney sections were washed twice with Tris-buffered saline (TBS), fixed with 4% formaldehyde for 20 min, permeabilized using 0.25% Triton X-100 for 30 min at room temperature, and incubated with blocking buffer containing 1% BSA for 15 min. Immunolabeling was conducted using primary antibodies against FN (1:800), and Col IV (1:500) at room temperature for 1 h followed by FITC-conjugated goat anti-rabbit secondary antibody. Signals were observed by fluorescence microscopy (IX71; Olympus, Tokyo, Japan) and quantified using Image J.

### Immunohistochemical (IHC) Staining

Kidney sections were immunohistochemically stained as described (23). Briefly, 4-μm-thick kidney sections were incubated with anti-FN (1:500), anti-Col IV (1:500), anti-STAT1 (1:500), and anti-TGFβ1 antibody (1:150) overnight at 4°C, followed by incubation with horseradish peroxidase-conjugated anti-rabbit secondary antibody (BBI Life Science, Shanghai, China). The sections were counterstained with hematoxylin after being developed with diaminobenzidine to produce a brown colored precipitate. The sections were analyzed by light microscopy.

### Quantitative Reverse Transcriptase Polymerase Chain Reaction (qRT-PCR)

Total RNA was extracted from cultured HK-2 cells or renal tissues using TRIzol reagent (Takara, Japan) according to the manufacturer's protocols. cDNA was synthesized using AMV First Strand cDNA Synthesis Kit (Shanghai Biological Engineering, China). Amplification reactions were carried out using SGExcel Ultra SYBR Mixture (Shanghai Biological Engineering, China). qPCRs were run in a 7,500 Fast Real-Time PCR System (Applied Biosystems, Foster City, CA, USA). The 2^−ΔΔCt^ method was used for relative mRNA quantification using *GAPDH* as an internal control. All experiments were performed in triplicate. Primer sequences are listed in Table 2.

**Table 2:** Sequences of primers used for quantitative RT-PCR analysis.

**Gene symbol**	**Forward primer(5′-3′)**	**Reverse primer(5′-3′)**
Mouse GAPDH	GGTTGTCTCCTGCGACTTCA	TGGTCCAGGGTTTCTTACTCC
Human GAPDH	CAGGAGGCATTGCTGATGAT	GAAGGCTGGGGCTCATTT
Mouse STAT1	GATGGAGCTTGACGACCCTA	TCCTCTGGAGACATGGGAAG
Human STAT1	TGATGGCCCTAAAGGAACTG	CCGAGACACCTCGTCAAACT
Mouse TGFβ1	GCTGAACCAAGGAGACGGAA	GTTTGGGGCTGATCCCGTT
Human TGFβ1	TGGACATCAACGGGTTCACTA	GAAGCAGGAAAGGCCGGTT
Mouse FN	CTGCGCCCTACTCTCCTGTT	CTCATACTCCACCCGGAAGC
Human FN	ACCAGCACACCTGTGACCAG	GACCCAGGAGACCACAAAGC
Mouse Col IV	CAAAGGCTCCAAGGGTGAAG	GCCAGGTAAGCCAGGTTGTC
Human Col IV	GAGAAAGGTGAACCCGGAAA	CGGCCTATGAGTCCTGGGTA

*STAT1, signal transducers and activators of transcription-1; FN, fibronectin; Col IV, collagen type IV*.

### Western Blot Analysis

Total protein lysates from renal cortex and HK-2 cells were prepared using RIPA lysis buffer (ComWin Biotech, Beijing, China) with 1% protease and phosphatase inhibitors. Protein concentration was determined using a BCA assay (Dingguo, Beijing, China) according to the manufacturer's protocol. Lysates containing 20–50 μg of protein were separated by 8–12% sodium dodecyl sulfate polyacrylamide gel electrophoresis (SolarBio, Beijing, China) and transferred to polyvinylidene fluoride membranes. The membranes were blocked with 5% non-fat milk in TBS-T before incubation with primary antibodies specific for β-actin (1:1,000) used as the control, STAT1 (1:1,000), TGF-β1 (1:1,000), Col IV (1:500), and FN (1:500) overnight at 4°C with gentle shaking. The membranes were washed 3 times (10 min each time) with TBS-T, and incubated with goat anti-rabbit secondary antibody (1:10,000) at room temperature for 1 h. Densitometry was carried out using Image J.

### Statistical Analysis

Data are presented as the mean ± SEM.Comparison of two groups in patients was performed by unpaired *t*-tests. One-way ANOVA with Bonferroni *post-hoc* test was used for multiple data comparison in HK-2 cells and diabetic mice. Statistical analysis was performed using GraphPad Prism 5.0 (GraphPad Software Inc., USA). *P* < 0.05 were considered statistically significant.

## Results

### Renal STAT1, TGFβ1, FN, and Col IV Expression in Patients With Diabetic Kidney Disease

**Figure 1:**
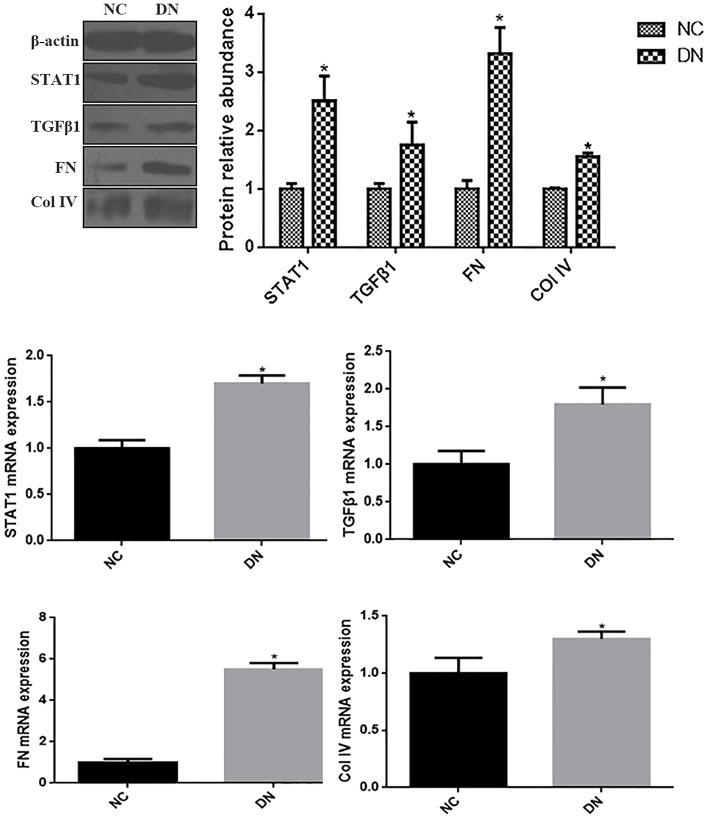
The expressions of STAT1, TGFβ1, FN, and Col IV in non-diabetic adjacent normal kidney tissues (NC), and renal biopsy sections from DN patients. **(A)** Protein expression levels of STAT1, TGFβ1, FN, and Col IV were quantified by western blot analysis. **(B)** mRNA expressions levels of STAT1, TGFβ1, FN, and Col IV were quantified by qRT-PCR. *P < 0.05 vs. NC.

**Figure 2:**
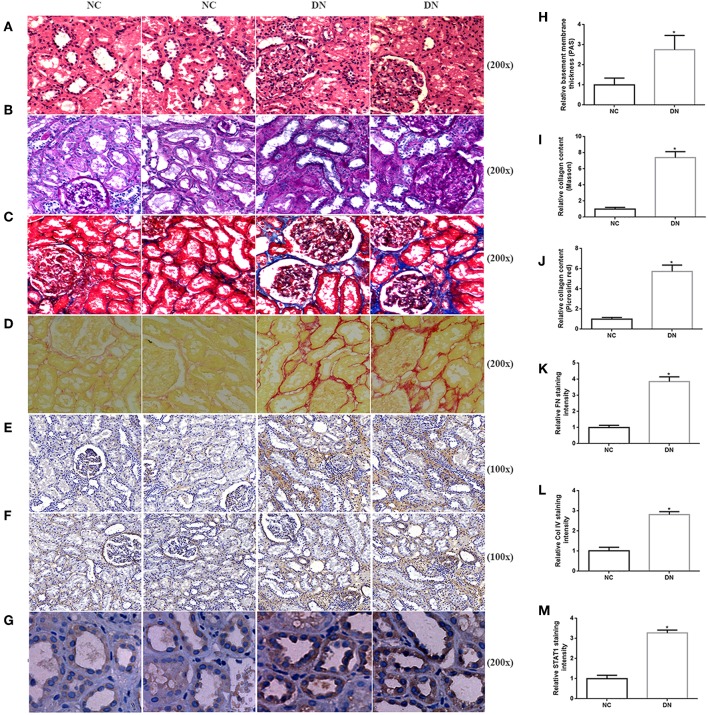
Histopathological examination of non-diabetic adjacent normal kidney tissues (NC) and renal biopsy sections from DN patients. Representative images of **(A)** HE staining, **(B)** PAS staining, **(C)** Picrosirius red staining, and **(D)** Masson's trichrome staining for assessing TIF. Immunohistochemistry staining of **(E)** FN, **(F)** Col IV, and **(G)** STAT1 in human kidney sections. Original magnification, 100–200x. Quantitative analysis of **(H)** PAS staining, **(I)** Picrosirius red staining, **(J)** Masson's trichrome staining, **(K)** relative FN staining intensity, **(L)** relative Col IV staining intensity, and **(M)** relative STAT1 staining intensity. All data are expressed as means ± SEM, *n* = 10 (*P < 0.05 vs. NC).

As indicated by qRT-PCR and western blot analyses of kidney biopsy tissue, expression of renal FN and Col IV, as well as STAT1 and TGFβ1 was significantly higher in renal tissue from patients with DN than in control tissue obtained from non-diabetic subjects (Figure 1), the whole western blots seen in the Supplementary Figure 1. Changes in tubulointerstitial morphology and TIF were evaluated by histological analysis of kidney biopsies obtained from DN patients and in control tissue. H&E and PAS staining revealed glomerular mesangial expansion and tubular atrophy in DN patients (Figures 2A,B,H). Moreover, Picrosirius red and Masson's trichrome staining demonstrated exacerbated renal TIF manifested by extracellular collagen deposition in renal biopsies from DN patients (Figures 2C,D,I,J). IHC staining also showed that the protein levels of STAT1, FN, and Col IV were increased in the tubulointerstitium of patients with DN as compared to control tissue (Figures 2E–G,K–M).

### Effects of Dapagliflozin on Metabolic Parameters in Diabetic Mice

The therapeutic potential of DAPA was investigated in STZ-induced diabetic mice. As expected, glucose levels were significantly higher in diabetic mice than the normal, non-diabetic, control group (Table 3, *P* < 0.05). The 24 h urine volume and proteinuria were substantially higher in diabetic mice than in normal controls, with the levels in diabetic mice increasing in a time-dependent manner from 8 weeks to 16 weeks, while DAPA administration for 16 weeks declined 24 h urine volume and proteinuria by 283% (not shown) and 200% (Table 3), respectively. Besides, DAPA significantly reduced blood glucose levels in mice in the DAPA-16W group compared with the DM-16W mice treated with saline (Table 1, *P* < 0.05). Serum BUN and serum creatinine were significantly increased in diabetic as compared to control mice. After 16 weeks of DAPA treatment, BUN and serum creatinine levels were significantly decreased with 37 and 32%, respectively (Table 3). Fasting body weight was lower in the diabetic mice than in the normal control mice, while kidney/body weight ratio was increased in diabetic mice. Administration of DAPA in diabetic mice did not decrease fasting body weight, although a significant decrease in kidney/body weight ratio was observed (Table 3).

**Table 3:** Influence of dapagliflozin on renal functional parameters in diabetic mice.

	**NC**	**DM-8W**	**DM-12W**	**DM-16W**	**DM-16W+DAPA**
BW (g)	23.3 ± 1.36	20.8 ± 2.02^*^	18.8 ± 1.63^Δ^	17.6 ± 0.56^δ^	19.0 ± 1.99^#^
BG (mmol/l)	6.63 ± 0.86	25.7 ± 4.74^*^	28.8 ± 1.15^*^	29.9 ± 2.34^*^	17.9 ± 3.16^#^
Scr (mg/dL)	8.67 ± 2.08	15.3 ± 2.52^*^	22.3 ± 6.11^Δ^	32.1 ± 4.00^δ^	21.7 ± 4.04^#^
24 h Pro (g)	0.39 ± 0.23	1.28 ± 0.15^*^	2.10 ± 0.28	2.69 ± 0.26^δ^	1.29 ± 0.10^#^
Relative kidney weight (mg/g)	5.13 ± 0.62	7.79 ± 0.82^*^	9.65 ± 1.22^Δ^	11.5 ± 1.33^δ^	8.34 ± 0.41^#^
BUN (mg/dl)	6.47 ± 1.25	8.56 ± 0.19^*^	15.8 ± 0.95^Δ^	21.4 ± 1.20^δ^	13.5 ± 1.56^#^

BW, body weight; BG, blood glucose; Scr, serum creatinine; 24 h Pro, 24 h proteinuria; BUN, blood urea nitrogen.

* P < 0.05 vs. NC;

Δ P < 0.05 vs. DM-8W;

δ P < 0.05 vs. DM-12W;

# *P < 0.05 vs. DM-16W. Values are means ± SEM*.

### Dapagliflozin Suppresses STAT1/TGF-β1 Signaling and TIF in Diabetic Mice

**Figure 3:**
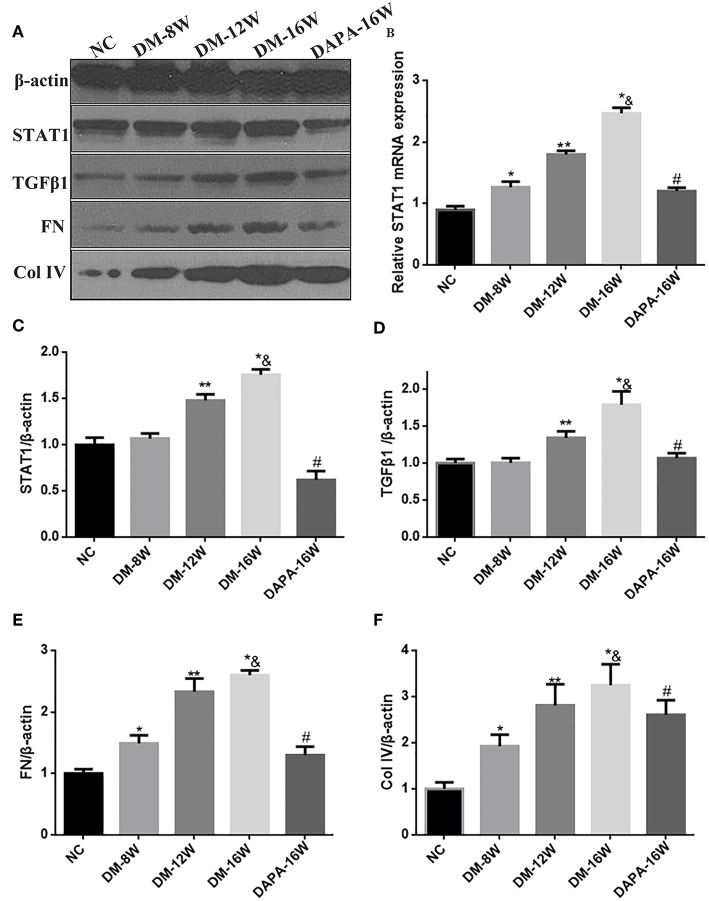
Effect of DAPA on renal production of fibrotic markers in diabetic mice. **(A)** Representative western blots for STAT1, TGF-β1, Col IV, and FN in renal cortical lysates. β-actin was used as internal control. **(B)** Density ratio of STAT1 to GAPDH mRNA expressions. **(C–F)** Normalized quantification of western blot data in diabetic mice at different times. *P < 0.05 vs. NC; **P < 0.05 vs. DM-8W; * ^&^*P* < 0.05 vs. DM-12W; ^#^*P* < 0.05 vs. DM-16W. Values are means ± SEM. *n* = 3.

**Figure 4:**
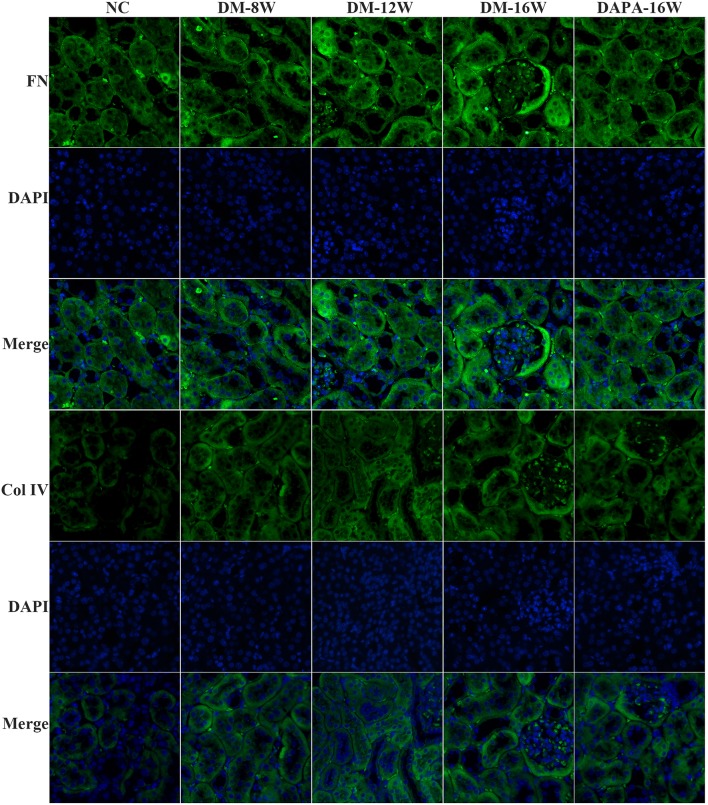
DAPA prevents renal TIF in diabetic mice. Representative images of immunofluorescence staining for Col IV and FN in tubular areas of diabetic mice. Original magnification, 200×.

**Figure 5:**
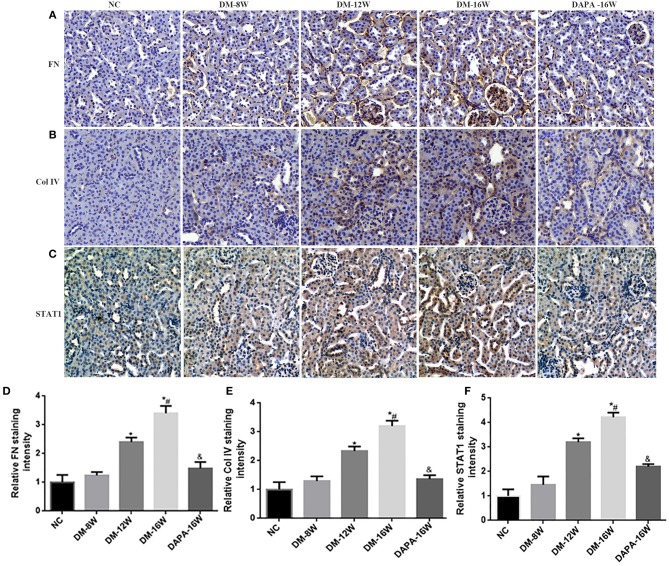
Effect of DAPA on extracellular matrix (ECM) protein accumulation and renal TIF. **(A–C)** IHC examination of kidney sections for FN, Col IV, and STAT1. Original magnification, 200×. **(D–F)** Quantification of FN, Col IV, and STAT1 staining. *P < 0.05 vs. NC; ^#^*P* < 0.05 vs. DM-12W; ^&^*P* < 0.05 vs. DM-16W. Values are means ± SEM.

We first analyzed the time course of STAT1 and TGF-β1 expression during the progression of DN. During follow-up in diabetic mice kidney protein levels of STAT1 and TGF-β1 (Figures 3A,C,D) were significantly increased in a time-dependent manner along with the development of DN. DAPA treatment in diabetic mice for 16 weeks dramatically reduced the expression of STAT1 and TGF-β1 compared with saline treatment for 16 weeks in diabetic mice, and normalization to non-diabetic control levels was observed (Figures 3A,C,D). Overproduction of extracellular matrix is a hallmark of DN and leads to renal interstitial fibrosis. The results showed that Col IV and FN protein levels were also time-dependently increased (similar to STAT1 and TGF-β1) as demonstrated by western blotting (Figures 3A,E,F). DAPA treatment for 16 weeks significantly reduced Col IV and FN protein expression (Figures 3A,E,F). Western blot data were qualitatively confirmed by immunofluorescence (Figure 4) and IHC showing robust increases in STAT1, Col IV and FN protein expression in the tubulointerstitium of diabetic mice with a significant reduction in diabetic mice treated with DAPA for 16 weeks (Figures 5A–F).

**Figure 6:**
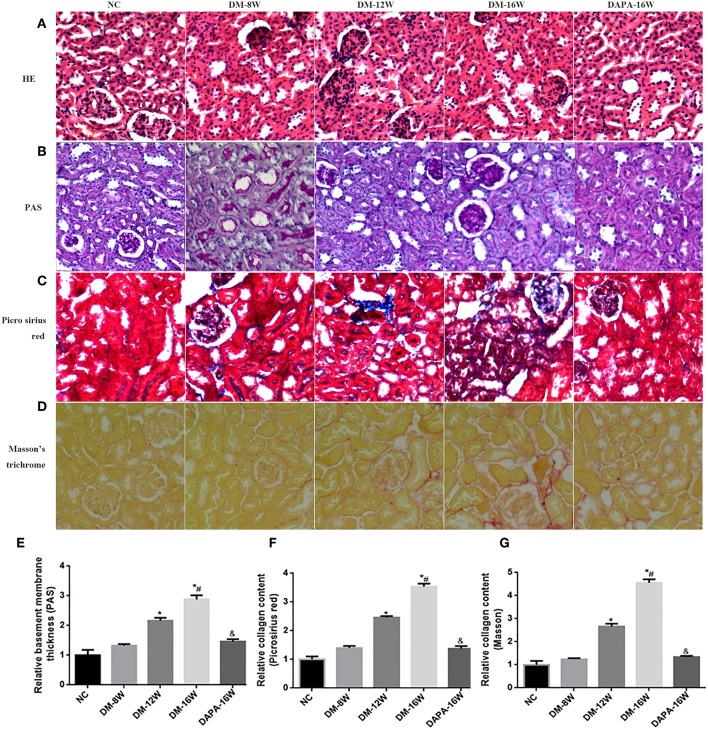
Effect of DAPA on renal TIF. **(A–D)** Representative photomicrographs of H&E, PAS, Picrosirius red, and Masson's trichrome staining. (**E–G**) Quantification of PAS, Picrosirius red and Masson's trichrome staining. *P < 0.05 vs. NC; ^#^*P* < 0.05 vs. DM-12W; ^&^*P* < 0.05 vs. DM-16W. Values are mean ± SEM.

Histologic assessment of PAS-stained renal tissue revealed that DAPA treatment in diabetic mice attenuated several morphologic changes related DN, including reduced tubular atrophy and TIF (Figures 6B,E). H&E staining further demonstrated that the tubular basement membrane was increased in diabetic mouse kidneys (Figure 6A). DAPA treatment reduced the deposition of ECM as compared with diabetic mice treated with saline. Diabetes induced a time-dependent increase in tubulointerstitial ECM deposition (i.e., TIF) as indicated by increased Picrosirius red (Figures 6C,F) and Masson staining (Figures 6D,G) when compared with normal control mice. Treatment with DAPA resulted in a marked decrease in TIF in diabetic kidneys.

### Dapagliflozin and Fludarabine Alleviate High Glucose-Induced STAT1/TGFβ1 Expression and EMT in HK-2 Cells

**Figure 7:**
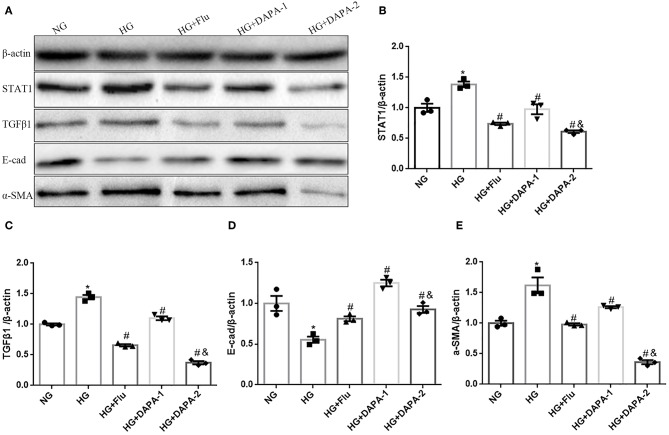
Effects of DAPA and Flu on the expression of EMT-related proteins in HK-2 cells. **(A)** Representative western blots illustrating STAT1, TGF-β1, E-cadherin, α-SMA, and β-actin protein expression in HK2 cells. **(B–E)** Quantification of the western blot data in HK-2 cells. Statistical analysis was performed with one-way ANOVA. Results are expressed as the mean ± SEM. *P < 0.05 vs. NC, ^#^*P* < 0.05 vs. HG, ^#&^*P* < 0.05 vs. HG+DAPA-1.

To corroborate the experimental *in vivo* model findings, we assessed *in vitro* the effect of DAPA and Flu on HK-2 cells stimulated with HG in an attempt to mimic the diabetic milieu. Exposure of HK-2 cells to HG induced cellular changes that are characteristic of EMT, including decreased expression of the epithelial marker E-cadherin and increased levels of α-SMA, as demonstrated by western blotting (Figures 7A,D,E) and RT-PCR (TGF-β1 stimuli as a positive control, seen in Supplementary Figure 2). However, both of DAPA and Flu could effectively reduce HG-induced over-expression of α-SMA (Figures 7A,E), but reversed the decreased expression of E-cadherin in a dose-dependent manner (Figures 7A,D). To explore the mechanism underlying the regulation of tubular epithelial cell EMT by DAPA, we examined the expression levels of STAT1 and TGFβ1 (i.e., factors known to promote EMT) by western blotting. Results indicate that HG, significantly increased STAT1 and TGF-β1 expression in HK-2 cells, which was strongly reduced after incubation with DAPA and Flu (Figures 7A–C).

## Discussion

DN is one of the most serious microvascular complications of diabetes, and one of the most important conditions influencing patients' quality of life and survival (26). In addition to current therapies, new molecular mechanisms and therapeutic approaches are needed. In the present study, we demonstrated that the selective SGLT2 inhibitor DAPA, lowered blood glucose levels and proteinuria, improved renal function as evidenced by reduced serum creatinine and BUN levels in diabetic mice. Beyond the improvement of renal function and metabolic outcomes, the main findings of the current study are the protective effects of DAPA on the development of renal TIF in diabetic mice. Our study revealed that DAPA successfully inhibited hyperglycaemia-induced ECM deposition in the kidneys of diabetic mice, attenuated EMT of tubular epithelial cells under hyperglycemic conditions, and suppressed the level of the profibrotic factors STAT1 and TGFβ1 both *in vivo* and *in vitro*.

Human renal biopsy data revealed that in renal tissues from DN patients, tubular expression of FN and Col IV were significantly increased with concurrent development of TIF as shown by ECM deposition using histochemistry. Furthermore, we found that the expression of STAT1 and TGFβ1 was significantly increased in patients with DN, entirely consistent with protein levels of FN and Col IV and severity of TIF in the development of DN. This may suggest a direct relationship among STAT1/TGFβ1 expression, ECM deposition and the severity of renal TIF. Although clinical outcome trials have shown that SGLT2 inhibitors delay the progression of DN and improve major outcomes in patients with T2DM, the precise underlying mechanism is still unclear.

Tang et al. (27) has demonstrated the protective effect of DAPA on the progression of renal fibrosis in a type 2 diabetic mouse model. However, the effect of DAPA on the renal tubulointerstitial injury in type 1 diabetes was not addressed. Nonetheless, the present study extends these findings in renal TIF in a model of type 1 diabetes in mice. We administered the selective SGLT2 inhibitor DAPA, to a well-established model of STZ-induced diabetes, and investigated its effects on the development of diabetes-induced renal TIF. Interestingly, our findings showed that the expression of Col IV and FN increased in a time-dependent manner in diabetic mice from 8 to 16 weeks. Similar results were found for the expression of STAT1 and TGFβ1. These changes indicative of TIF were accompanied by functional changes such as increased proteinuria, serum creatinine and BUN.

Compared with saline-treated diabetic mice for 16 weeks, we found that DAPA -treated diabetic mice had a significant decrease in in the expression of the profibrotic factors STAT1, TGFβ1, and ECM proteins Col IV and FN. These results illustrated that the expression of STAT1/TGFβ1 confers an unfavorable effect on renal function and might serve as an alternative approach to reflect the severity of renal TIF. Besides, we demonstrated that a 16 week exposure to DAPA effectively attenuated the hyperglyceamia-induced increase in renal of FN and Col IV and the expression of STAT1 and TGFβ1, indicating that it is possible that the anti-fibrotic effects of DAPA are mediated through the STAT1/TGFβ1 signaling pathway. The results demonstrated that DAPA improved hyperglycemia and delayed TIF development in diabetic mice, which is consistent with the previous finding that DAPA reduced interstitial fibrosis by acting directly on the interstitium in Akita mice (17). Gallo et al. (28) also demonstrated in the db/db mouse model of type 2 diabetes, the upregulation of some profibrotic genes in the kidneys was ameliorated upon SGLT2 inhibition by empagliflozin.

In the current study, the protective effects of DAPA on the kidneys paralleled the effects on lowering glucose levels, suggesting that reduction of glucose toxicity induced by DAPA, is considered to be an important mechanism for the observed restoration of the STAT1/TGFβ1 pathway.

In addition, studies on cardiac fibrosis have shown that DAPA is a potential anti-fibrotic drug for the prevention and treatment of cardiac fibrosis after myocardial infarction through glucose-independent effects (29). It was consistent with our studies in HG -induced EMT in HK-2 cells, EMT plays a significant role in renal TIF, which is one of the pathological hallmarks of DN. As a central component of the cell adhesion junction, E-cadherin is required for maintaining epithelial integrity. Loss of E-cadherin and excessive α-SMA are the most important aspects of EMT (30). Our results provided novel insight into STAT1-mediated EMT of renal tubular proximal epithelial cells under hyperglycemic conditions in HK-2 cells, which showed the beneficial effects of DAPA treatment on attenuation of STAT1 expression and tubular EMT appeared to be glucose-independence. Furthermore, we found that Flu significantly decreased the expression of STAT1 and TGFβ1 in HK-2 cells, which indicates a possible mechanism that DAPA ameliorates EMT by suppressing hyperglycemia-induced STAT1/TGFβ1 pathway. Previous studies have demonstrated that DAPA ameliorates DN by suppressing hyperglycemia-induced oxidative stress in Akita and ob/ob mice (17). To the best our knowledge, it is possible that DAPA suppress the JAK-STAT signaling cascade by targeting oxidative stress induced high glucose, and indicates a possible mechanism to explain the protective effect of DAPA in DN through STAT1/TGFβ1 signaling pathway, although additional studies are needed to delineate the detail mechanism underlying this process.

In conclusion, our study revealed that DAPA prevents TIF in kidneys of diabetic mice and attenuates EMT of tubular epithelial cells under hyperglycemic conditions. Mechanistically, the renoprotective and anti-fibrotic effects of DAPA are mediated, at least part, by the inactivation of the STAT1/TGF-β1 signaling pathway and target gene expression. Given the above, DAPA could be a useful therapeutic strategy to halt the onset and progression of renal TIF in DN. However, whether these beneficial effects of DAPA are solely related to the reduction in blood glucose or glucose-dependent pathways, the mechanism of DAPA influence the expression of STAT1 and TGF-β1 are required to further explore. There might be additional and synergistic direct protective renal effects beyond glucose lowering effects, but this remains to be further explored.

## Ethics Statement

Kidney biopsy sections from patients diagnosed with class III or IV DN (*n* = 10) and non-diabetic adjacent normal tissues from patients with renal cell carcinoma (NC, *n* = 10) were obtained from the first affiliated hospital of Zheng Zhou University following the Institutional Review Board of Zhengzhou University.

Animal experiments were approved by the institutional committees of the Animal Research Committee and Animal Ethics Committee of Zhengzhou University.

## Author Contributions

FH performed literature searches and established the mouse model of STZ-induced T1DM. YZ was responsible for blood glucose monitoring. LJ and TA performed western blotting. FH performed the qRT-PCR analysis and statistical analysis. QW was responsible for histological staining and morphological analysis. GQ designed the experiment and supervised the project. FH wrote the manuscript with contribution from all authors. J-LH and JB were responsible for critical reading, editing and revising the manuscript.

### Conflict of Interest Statement

The authors declare that the research was conducted in the absence of any commercial or financial relationships that could be construed as a potential conflict of interest.

## Supplementary Material

Supplementary Figure 1 The western blots for STAT1, TGFβ1, FN and Col IV in all human kidney samples.

Supplementary Figure 2 The mRNA expressions levels of STAT1, E-cadhein and a-SMA in HK-2 cells treated with TGF-β1 and high glucose. ^*^*P* < 0.05 vs. NG.
