# Supplementary figures and images for: Dapagliflozin Attenuates Renal Tubulointerstitial Fibrosis Associated With Type 1 Diabetes by Regulating STAT1/TGFβ1 Signaling
Source: Front Endocrinol (Lausanne). 2019 Jul 3;10:441. doi: 10.3389/fendo.2019.00441 (PMC6616082; doi:10.3389/fendo.2019.00441)

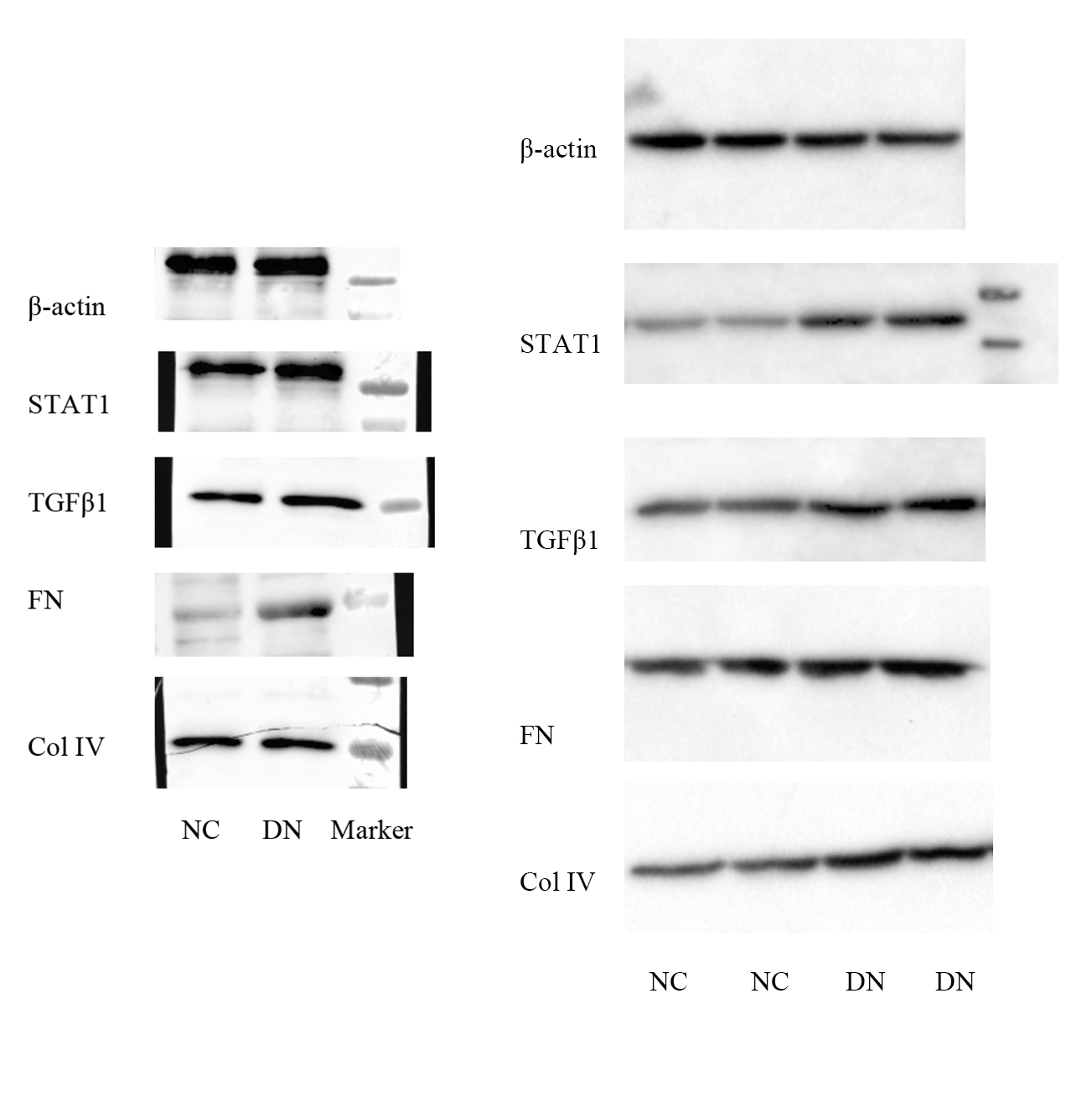

Supplement: Supplementary Figure 1 — The western blots for STAT1, TGFβ1, FN and Col IV in all human kidney samples. [file Image_1.TIF]

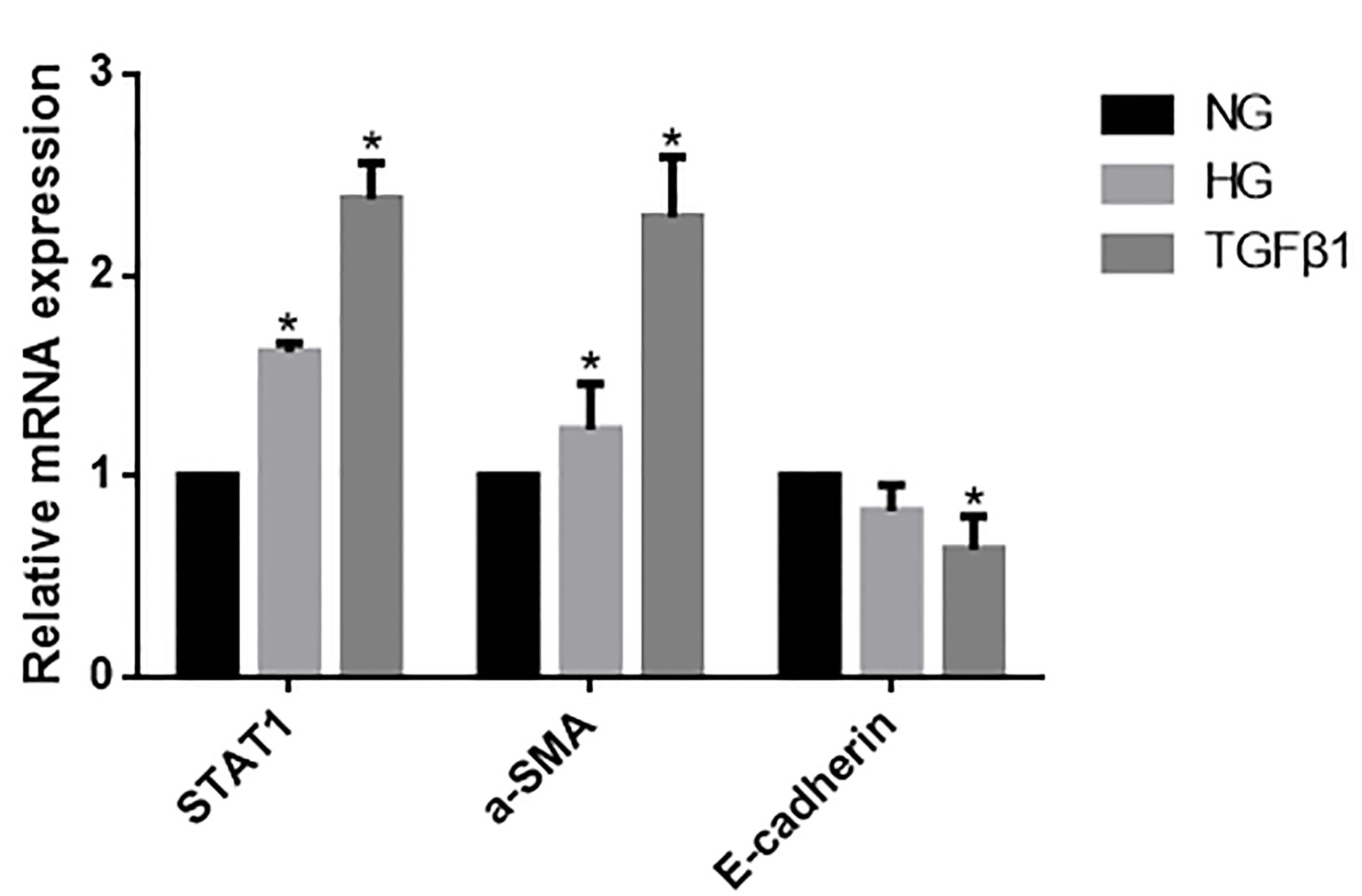

Supplement: Supplementary Figure 2 — The mRNA expressions levels of STAT1, E-cadhein and a-SMA in HK-2 cells treated with TGF-β1 and high glucose. *P < 0.05 vs. NG. [file Image_2.TIF]
